# Rationale for key elements of Sino-American collaboration in clinical research

**DOI:** 10.1186/1479-5876-10-S2-A4

**Published:** 2012-10-17

**Authors:** Robert M Califf

**Affiliations:** 1 Duke Translational Medicine Institute, Duke University, Durham, NC 27710, USA

## 

When new scientific discoveries are properly evaluated within the translational research continuum and definitive evidence is generated regarding the balance of benefits and risks associated with therapeutic interventions, dramatic health improvements are seen both for individuals and populations. Until recently, however, technological limitations dictated that definitive evidence could be generated for only a fraction of interventions under development, because clinical investigation was limited by geography and access to expertise. Today, less than 15% of major medical decisions are informed by high-quality evidence [1]. Furthermore, increasing knowledge about the roles played by genetics and practice environments demonstrates that we must develop multinational studies to generate evidence relevant to particular biological and cultural contexts [2]. It is now time to begin to plan for a global learning health system.

Modern informatics and information technology have enabled the sharing of research data without regard to geographic boundaries. These new capabilities expand the concept of human biomedical research from an activity conducted in a limited number of specialized centers to a global activity accessible to all patient populations and qualified practitioners [3]. With appropriate informatics support, shared protocols, and facilitative cultural elements, common diseases can be studied on a larger scale and clinical trials in rare diseases will be able to accrue adequate sample sizes, enabling valid inferences to be drawn. In essence, the limits on knowledge generation will henceforth be determined by two key factors: (1) the number of qualified individuals in the clinical and translational research workforce and (2) the degree to which regulatory and funding sources encourage broad-scale collaboration.

Examples of therapeutic areas where progress could be accelerated include diabetes (which affects global populations in large numbers), congenital heart disease (which affects 1% of all global populations) and Pompe disease (a rare disease with a new effective therapy). In each case, collaborative studies between China and the United States—two of the world’s largest funders of biomedical research—could provide the example to stimulate similar activity on a global scale.

We propose that five key programs specific to clinical and translational research [4] (Table 1) will need to train and educate a vast workforce over the coming decade in order to capitalize on these technological advances.

**Table 1:** Key programs for clinical & translational research

Clinical research training
Epidemiology and global health
Biostatistics
Medical informatics
Health sector management

## References

[B1] Tricoci P Allen JM Kramer JM Califf RM Smith SC Jr Scientific evidence underlying the ACC/AHA clinical practice guidelines JAMA 2009 301 831 841 Erratum in: JAMA 2009, 301:1544 10.1001/jama.2009.205 19244190

[B2] Califf RM Zarin DA Kramer JM Sherman RE Aberle LH Tasneem A Characteristics of clinical trials registered in ClinicalTrials.gov, 2007-2010 JAMA 2012 307 1838 1847 10.1001/jama.2012.3424 22550198 PMC13431740

[B3] Dzau VJ Ackerly DC Sutton-Wallace P Merson MH Williams RS Krishnan KR Taber RC Califf RM The role of academic health science systems in the transformation of medicine Lancet 2010 375 949 953 10.1016/S0140-6736(09)61082-5 19800111

[B4] CMS Innovation Health Care Innovation Awards: Project Profiles – Duke University http://innovation.cms.gov/initiatives/innovation-awards/project-profiles.html

